# Response to: Comment on “The Effects of Hemodialysis on Tear Osmolarity”

**DOI:** 10.1155/2016/1925683

**Published:** 2016-08-23

**Authors:** Muhittin Taskapili, Kubra Serefoglu Cabuk, Rukiye Aydin, Kursat Atalay, Ahmet Kirgiz, Dede Sit, Hasan Kayabasi

**Affiliations:** ^1^Prof. Dr. N. Resat Belger Beyoglu Eye Training and Research Hospital, 34421 Istanbul, Turkey; ^2^Bagcilar Training and Research Hospital, Ophthalmology Department, 34200 Istanbul, Turkey; ^3^Ophthalmology Department, Medipol University, 34214 Istanbul, Turkey; ^4^Bagcilar Training and Research Hospital, Nephrology Department, 34200 Istanbul, Turkey

We thank Onder Ayyildiz and Gokhan Ozge for their interest and comment on our paper “The Effects of Hemodialysis on Tear Osmolarity” [1, 2]. They thought that detection of TO would be performed at the same time of the day regarding the duration of hemodialysis (HD) which may avoid the bias of the methodology according to the study of Niimi et al. [3].

Niimi et al. have enrolled 38 medically healthy neophytes. Their subjects reported to the CRC an average of 14 ± 2.0 hours (7–17 hours) after awakening for baseline measurements and sleeping at the CRC, thereby allowing for uniform environmental exposure (e.g., humidity and temperature) and timely collection of measurements upon awakening.

The physical conditions of our clinic are not suitable for all HD patients to report 7 hours after awakening for baseline measurements and sleeping one day thereby allowing for uniform environmental exposure (e.g., humidity and temperature) and timely collection of measurements upon awakening.

In our Hemodialysis Unit, two HD sessions have been performed; the first session was between 8:30 and 12:30 and the second session was between 13:00 and 17:00. 23 patients were in the first session and 20 patients were in the second session in our study. Tear osmolarity (TO) measurements have been performed one minute before the beginning of HD and 30 minutes after the termination of HD. There was no statistically significant difference between pre-HD tear TO measurements and between post-HD TO measurements of these two sessions (Table 1).

## Figures and Tables

**Table 1:** Pre-HD and post-HD tear osmolarity according to HD sessions.

	First session	Second session	*p*
Pre-HD tear osmolarity	312.2 ± 16.6	315.6 ± 18.9	0.528
Post-HD tear osmolarity	301.6 ± 18.3	302.1 ± 12.3	0.929

Statistical method: Student's *t*-test independent.
